# Advances in the Resection and Reconstruction of Midfacial Tumors Through Computer Assisted Surgery

**DOI:** 10.3389/fonc.2021.719528

**Published:** 2021-10-19

**Authors:** Max Wilkat, Norbert Kübler, Majeed Rana

**Affiliations:** Department for Oral & Maxillofacial Surgery, University Hospital Düsseldorf, Düsseldorf, Germany

**Keywords:** midfacial tumors, computer assisted surgery, navigated resection, computer assisted reconstruction, orbital volume measurement

## Abstract

Curatively intended oncologic surgery is based on a residual-free tumor excision. Since decades, the surgeon’s goal of R0-resection has led to radical resections in the anatomical region of the midface because of the three-dimensionally complex anatomy where aesthetically and functionally crucial structures are in close relation. In some cases, this implied aggressive overtreatment with loss of the eye globe. In contrast, undertreatment followed by repeated re-resections can also not be an option. Therefore, the evaluation of the true three-dimensional tumor extent and the intraoperative availability of this information seem critical for a precise, yet substance-sparing tumor removal. Computer assisted surgery (CAS) can provide the framework in this context. The present study evaluated the beneficial use of CAS in the treatment of midfacial tumors with special regard to tumor resection and reconstruction. Therefore, 60 patients diagnosed with a malignancy of the upper jaw has been treated, 31 with the use of CAS and 29 conventionally. Comparison of the two groups showed a higher rate of residual-free resections in cases of CAS application. Furthermore, we demonstrate the use of navigated specimen taking called tumor mapping. This procedure enables the transparent, yet precise documentation of three-dimensional tumor borders which paves the way to a more feasible interdisciplinary exchange leading e.g. to a much more focused radiation therapy. Moreover, we evaluated the possibilities of primary midface reconstructions seizing CAS, especially in cases of infiltrated orbital floors. These cases needed reduction of intra-orbital volume due to the tissue loss after resection which could be precisely achieved by CAS. These benefits of CAS in midface reconstruction found expression in positive changes in quality of life. The present work was able to demonstrate that the area of oncological surgery of the midface is a prime example of interface optimization based on the sensible use of computer assistance. The fact that the system makes the patient transparent for the surgeon and the procedure controllable facilitates a more precise and safer treatment oriented to a better outcome.

## Introduction

Progress in the planning and implementation of computer assisted surgery (CAS) with the aid of 3D images for the area of the jaw and facial tumors has been recorded over the past two decades, particularly in order to optimize the interface-supporting procedure in tumor surgery (1, 2). Especially during staging, computer-assisted planning and multi-planar as well as -modal evaluation and analysis of data (CT, MRT) have become indispensable and represent an essential pillar of tumor surgery and tumor management.

The complex 3D structures of the face and head require a lot of experience and skill on the part of the surgeon. Without computer-assisted planning and navigation, therapy is only possible with difficulty (3–5). Tumor resection is often associated with the loss of essential, functional structures of the face (6). To reconstruct the affected areas of the face, patient-specific autogenic or alloplastic solutions are used. Computer-assisted systems are increasingly used today to determine the optimal anatomical position and for navigation (7). These systems make use of the possibility of visualizing tumors and their boundaries in CT data as well as planning the reconstruction on virtual three-dimensional models, which delivers very good results in individual planning. However, the planning effort and the costs of previous systems are still high and additional tools and engineering know-how are required to perform the segmentation in the various data sets (MRT, CT, CBCT) manually and in a complex manner. In addition to multimodal image analysis, modern image analysis platforms allow the automatic segmentation of tumors (8, 9), the extension of this segmentation by a defined safety distance, the simulation of the bony reconstruction, the import of preformed three-dimensional reconstructions such as titanium grid structures, as well as the image fusion of the planning with the postoperative result (1, 10). It is suitable for preoperative analysis as well as intraoperative, interactive image information exploitation (navigation, intraoperative CT/CBCT) as well as for connection to other interfaces, such as postoperative tumor staging or radiation planning.

In our study we focused on application possibilities of CAS for oncologic surgery of the midface. These anatomically complex tumors require a highly sophisticated treatment planning in terms of resection and reconstruction. This is enabled through the usage of CAS. Moreover, CAS might contribute to a better interdisciplinary networking and an improved quality of life for the patient.

The task of the present work was therefore to evaluate to what extent oncological surgery of the midface can benefit from the use of CAS and in which areas it is superior to conventional therapy.

## Materials and Methods

### Study Design

This prospective clinical study was approved by the local ethics committee at the Hannover Medical School, Germany. The patient collective considered in this work was treated within the period from 2011 to 2019 in the Department for oral and maxillofacial surgery at the Hannover Medical School and at the University Hospital Düsseldorf.

31 patients with tumors of the upper jaw were treated through computer-assisted tumor resection including tumor mapping and primary reconstruction. A retrospectively analyzed patient sample of 29 cases which were treated through conventional methods without the use of computer assisted surgery served as a control group. The patients of the control group were selected with regard to tumor sizes and tumor locations that were comparable with the random sample of 31 patients that had been treated using CAS.The two groups were compared with regard to descriptive data, resection results and quality of life.

Due to the tumor extent, 15 of the above mentioned 31 patients had defects involving the orbit which needed primary orbit reconstruction. These 15 cases of primary reconstruction of the midface involving the orbit were evaluated for the pre- and postoperative volume of the orbit.

### Conventional Tumor Resection

For conventional tumor resection the surgeon evaluated the tumor extent on basis of the clinical findings involving inspection and palpation and the contemplation of the preoperative standard imaging procedure. The resection border was chosen to include a safety margin of 1 centimeter. Afterwards, the resection was performed followed by taking of circular resection-bed driven specimen of the mucosal resection margin and the deep resection margin for frozen section control. The mucosal resection margin was removed with a minimum of two specimen, while the deep resection margin was removed with a minimum of 1 specimen. In cases of extraoral skin resection, the skin resection margin was collected with a minimum of 2 specimen. The anatomical orientation of these taken resection margins were marked by indicating sutures whose orientation was written into the accompanying pathology request document. In case of positive frozen sections further resection was performed in the area indicated by the pathologist. The final resection status which also involved the bony margins was described in the definitive pathology report.

### Computer Assisted Surgery - Digital Workflow

**Figure 1:**
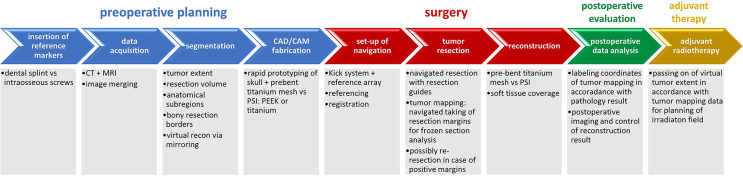
Complete workflow of computer-assisted surgery. Computer-assisted surgery involving preoperative planning with data acquisition and processing including segmentation and virtual resection and reconstruction as shown in blue, surgery with real-time navigation, resection with tumor mapping and reconstruction as shown in red, postoperative evaluation as shown in green. Except for adjuvant radiotherapy as shown in yellow, the illustrated workflow has been used as clinical routine for treatment of midface tumors.

The whole workflow of data acquisition, preoperative planning, tumor resection and mapping using intraoperative real-time navigation as well as reconstruction of midfacial defects involving the orbit after ablative surgery using CAD/CAM is demonstrated (see **Figure 1**).

### Data Acquisition and Preoperative Planning

**Figure 2:**
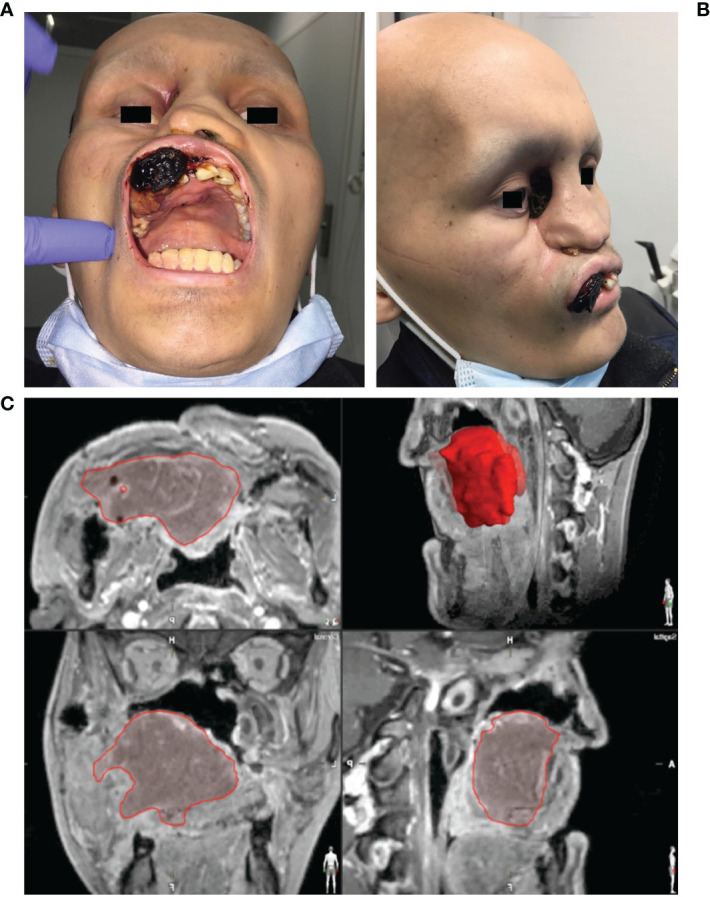
Clinical findings and virtual data in a case of Ewing’s sarcoma of the midface. **(A)** Frontal/intraoral and **(B)** side view of a 19-year-old patient with an Ewing’s sarcoma of the midface presenting with an ulcerating neoplasm in the area of the hard palate, the maxillary vestibule and the right medial nasal slope which has lead to a sinking of the right eyeball. **(C)** Segmented tumor extent (red) in the multi-planar view of the MRI images.

The process of digital planning was carried out using the planning software iPlan^®^ (version 3.0.5, Brainlab, Feldkirchen, Germany). With regard to intraoperative navigation-supported tumor mapping and tumor resection, imaging was acquired first (see **Figure 2**).

Imaging data was collected following a standard procedure for all cases. CT data was acquired during arteriovenous phase using contrast medium (field of view 20 cm, pitch 1.0, slice thickness 1.0 mm, 140-160 mA, pixel density 512 × 512). MRI imaging was additionally collected (T1- and T2-weighted images, 1.5T (1T = 800 kA/m), slice thickness 2 mm, pixel density 512 × 512) and merged *via* iPlan^®^ software (version 3.0.5, Brainlab, Feldkirchen, Germany) using the automatic ‘best fit’ algorithm. After automated fusion the result was manually checked in a multi-planar view considering the axial, coronal and sagittal planes. Conformation of best fit was indicated by manually accepting the result.

**Figure 3:**
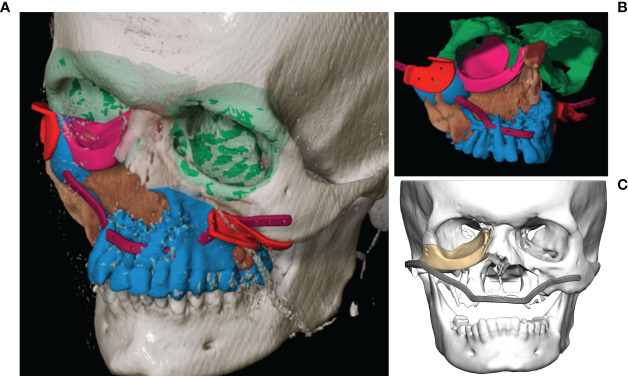
Virtual segmentation and planning of the functional defect reconstruction in a case of Ewing’s sarcoma of the midface. **(A, B)** Segmentation of the tumor extent (brown) and the planned bony resection (blue) with the resection border indicated by the cutting guides (red). The anatomical subunit of the left orbit has been mirrored to reconstruct the right orbit (both green) as a planning template for the right orbital floor reconstruction with a patient specific PEEK implant (pink). The outer contour of the maxilla is reconstructed with a patient specific titanium reconstruction plate (purple). **(C)** Both PSIs are displayed.

**Figure 4:**
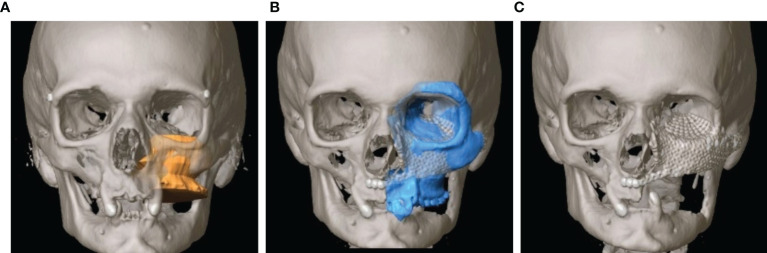
Virtual reconstruction planning using the principle of mirroring. **(A)** Segmented tumor of the left maxillary sinus with infiltration into the left floor of the orbit (yellow). **(B)** Template of the healthy right midface mirrored to the left (blue). **(C)** Postoperative CT shows the reconstruction of the orbit and the midface using two PSIs.

As soon as the image data were available in the required format, segmentation followed (see **Figure 3**). Segmentation was performed by the same senior surgeon as investigator for all 31 cases. In particular, the extent of the primary tumor was segmented, and the area of resection was defined by adding a three-dimensional safety margin in a freely selectable distance. Safety margin was set at 1 centimeter. On the basis of the segmented resection volume the bony resection margins were defined and used for designing resection guides. Moreover, anatomical subunits were segmented such as the bony orbit and the alveolar process of the upper jaw which both could be mirrored from the unaffected site for reconstruction purposes if applicable (see **Figures 3**, **4**).

### Set Up of Intraoperative Navigation

During the acquisition of the CT data set, either an individual dental splint with 4 additional titanium mini screws or in cases of a reduced or loosened set of teeth or planned resection which involved more than half of the dental arch in the upper jaw four surgically inserted 2.0 cross-drive titanium mini screws (DePuy Synthes^®^) have been used as reference markers during the individual registration of the patient.

**Figure 5:**
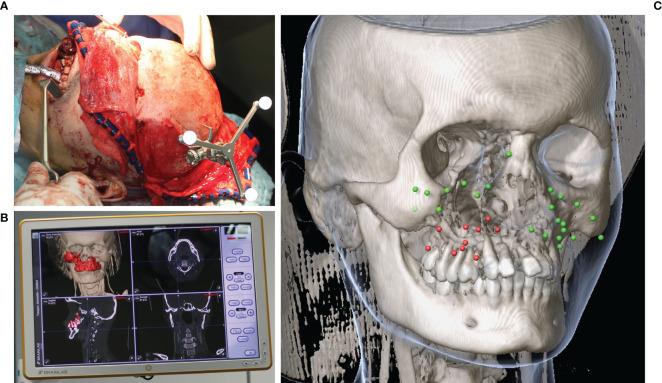
Intraoperative navigation during midfacial tumor ablation in a case of Ewing’s sarcoma of the midface. **(A)** Skull reference array attached for intraoperative navigation after coronal approach. **(B)** Real time navigation monitor showing the planned bony resection segmented in red color. **(C)** Data post-processing of the landmarks set intraoperatively after receipt of the histopathological report (green = tumor-free, red = tumor residual).

Before the operation, the patient was placed in a headrest. The reference star was fixed on the skull *via* the “Skull Reference-Array” plus osteosynthesis (see **Figure 5A**). The reference star was identified by the infrared camera of the navigation system Kick^®^ (Brainlab, Feldkirchen, Germany). identifying the exact position of the patient during operation. As mentioned above the previously inserted reference markers (a dental splint with additional titanium mini screws or titanium mini screws inserted to the skull) were navigated to in order to match their position to the coordinates of the reference markers previously saved in the data record. Additionally, a landmark test was carried out in order to compare the patient on the operating table with the virtual image in the data set and ensure that referencing has been successful.

The time for intraoperative navigation set-up has been measured to evaluate the mean surgery prolongation.

### Navigation-Guided Tumor Resection and Tumor Mapping

**Figure 6:**
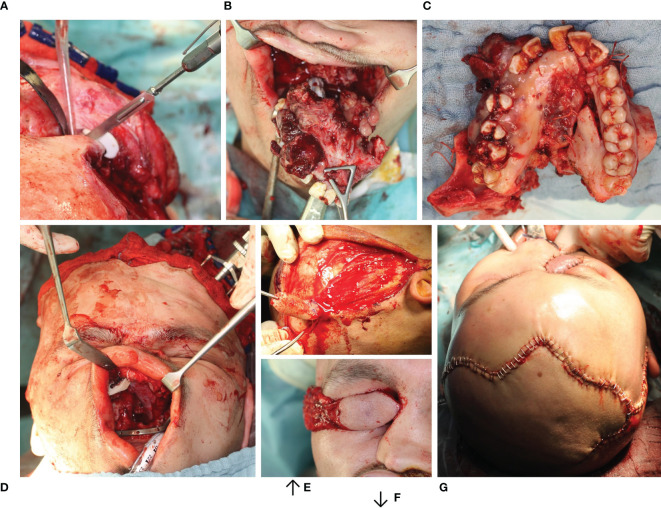
Resection of Ewing’s sarcoma of the midface with primarily functional reconstruction. **(A)** Bone resection using a cutting guide. **(B, C)** Tumor resection en bloc preserving the eyeball. **(D)** Primarily functional reconstruction reconstruction of the zygomatic bone and orbit floor using two patient specific implants (combined SLM titanium and PEEK (KLS Martin^®^, Tuttlingen) using the virtually mirrored left orbit as a template) both visible intraorally. **(E, F)** Dissection of the temporal muscle flap. **(G)** Wound closure with temporal muscle flap sewn in.

The treatment of midfacial tumor patients involving the tumor resection and the reconstruction was performed by only one team with the same senior surgeon being in charge during all CAS operations. Using the navigation probe, the extent of the virtually planned resection could be transferred to the intraoperative situation. Moreover, resection guides indicated the virtually planned resection margins concerning the bony margin (see **Figure 6A**). After navigated resection of the tumor (see **Figures 6B, C**), the resection-bed driven margins for frozen section analysis were collected with the aid of navigation by setting intraoperative landmarks with the help of the iPlan software (Version 3.0.5). At least 12 specimens were taken from the mucosal resection margin following the clockwork technique accompanied by at least four specimens from the deep resection margin. In cases of extraoral skin resection further specimen were taken following the clockwork technique. Coordinates of every three-dimensional position of a taken specimen were saved in the software. All these coordinates were thereby integrated into the virtual data to determine the exact three-dimensional position of the taken specimen of the resection margin. Moreover, screenshots of every coordinate were acquired, printed out and send to the pathologist accompanied by the specimen and the pathology request document to pass on the three-dimensional localization of the taken specimen. The information of specimen position has been linked to the pathology result of the corresponding specimen later on by color-coding: red meaning the specimen was positive for tumor residual, green meaning the specimen was tumor-free (see **Figure 5**).

In order to evaluate whether the method of intraoperative navigation-assisted tumor resection and mapping was successful in the sense that more complete resections could be carried out, tumor resection of the upper jaw with CAS were compared with the control group. Resection status was defined by the final report of the pathologist. This report considered the main tumor resection specimen - including the bony margins which needed more time for processing compared to soft tissue margins due to the necessary decalcification - as well as the taken resection margin specimen for frozen section analysis. It was differentiated between complete resection (R0), microscopically residual tumor mass (R1) and macroscopically residual tumor mass (R2).

### Primary Reconstruction After Ablative Surgery

The analysis of the tumor and resection margins showed the extent to which the reconstruction was restricted. Midface reconstruction true to original using titanium mesh structures or PEEK implants with soft tissue transplants was the aim (see **Figures 6D–G**). With unilateral tumor growth the virtual mirroring of the opposite healthy side served as a reference point for the reconstruction of the shape and the volume of the orbit true to the original so that not only the functional but also the aesthetic restoration was achieved (see **Figure 4**). The virtual reconstruction planning was exported as a stereolithography file (STL format) and printed out three-dimensionally or milled *via* “rapid prototyping” to create a bio-model made of cheap composite materials, which could also be autoclaved. On the basis of the virtual model a patient-specific 3D implant was pre-formed by cold forming titanium mesh. Alternatively, a PEEK implant or a titanium implant was manufactured *via* additive CAD/CAM procedure by KLS Martin (Tuttlingen, Germany) (see **Figure 3**). During the intraoperative navigation, the reference probe was used to check whether the implant has been inserted anatomically correctly, since the data set acted as a virtual template. For pre-bent titanium meshes the thickness of the mesh itself had to be considered. Therefore an offset according to the used mesh thickness has been used during navigational reconstruction control. In cases of CAD/CAM processed implants the STL-file of the implant itself was integrated into the data set for navigation control.

**Figure 7:**
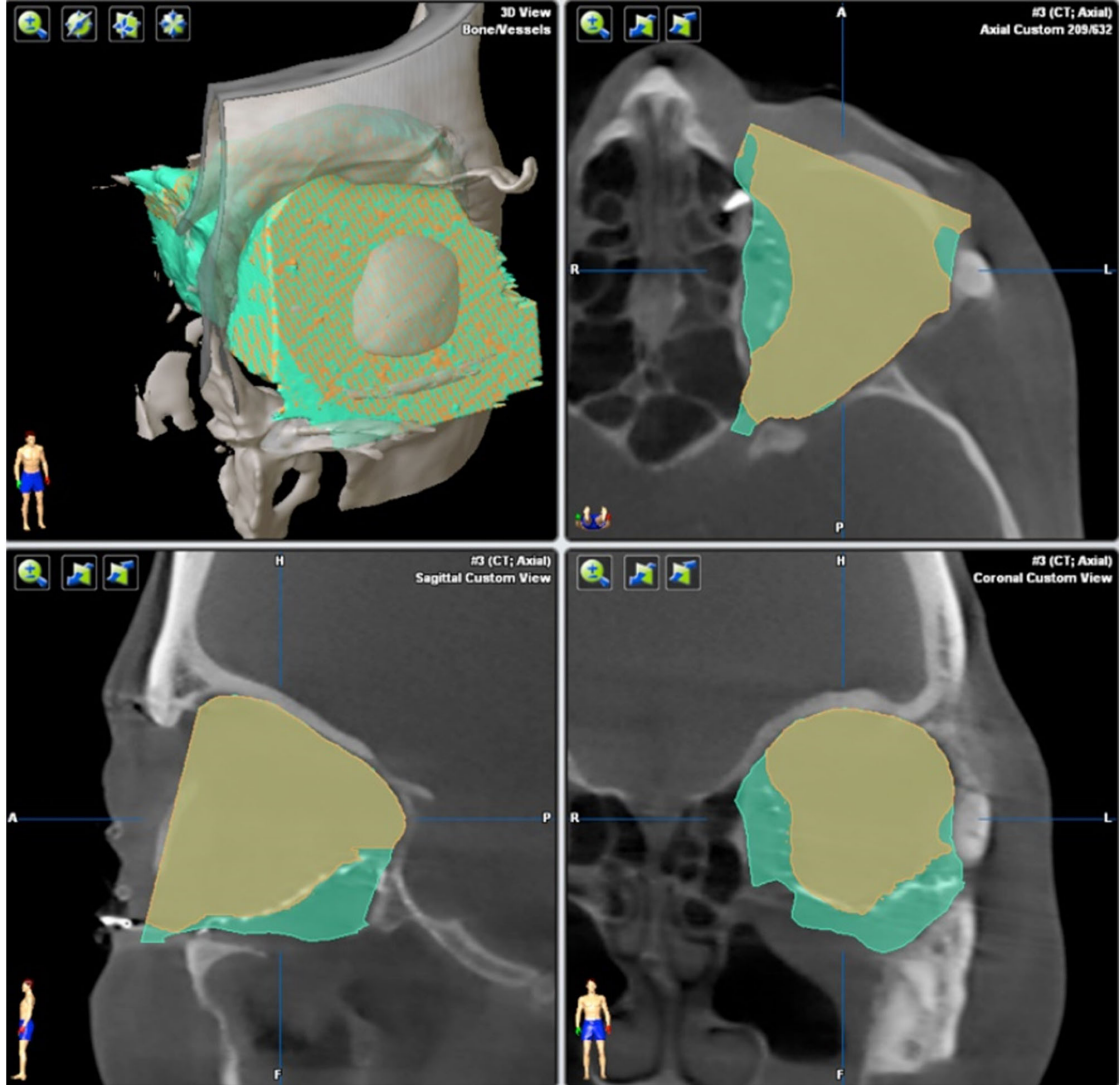
Calculation of orbit volume. Orbital cavity algorithm calculates the orbit volume not only within the bony boundaries (green), but also on the adjacent orbit 3D mesh (yellow). The volume calculation works automatically.

The software iPlan 4.0 beta was used to measure the intra-orbital volume. It is able to virtually subtract a pre-formed cuboid with a defined volume from the segmented bony boundaries so that the volume can be calculated. This algorithm is integrated in the currently available software and enables the surgeon to calculate the volume by simply clicking on the “Orbital cavity” button (see **Figure 7**).

**Figure 8:**
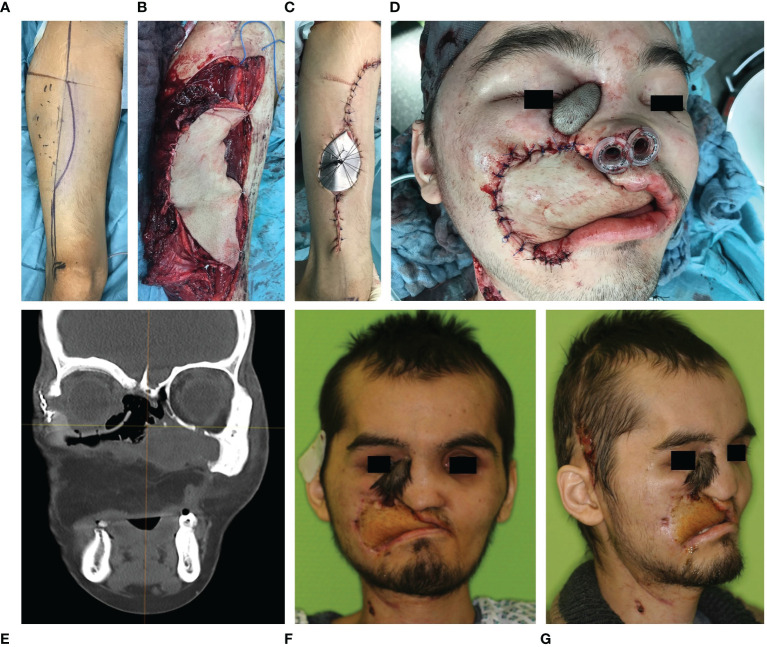
Early secondary soft tissue reconstruction of the upper lip and jaw using an anterolateral thigh flap in a case of Ewing’s sarcoma of the midface. **(A–C)** Skin marking, flap harvest and wound closure of the anterolateral thigh flap of the right lower extremity. **(D)** Anterolateral thigh flap sewed in. **(E)** Postoperative CT scan in coronal plane shows the patient specific implant *in situ* reconstructing the right orbital floor while the symmetrical position of the eyeball has been restored. **(F)** Frontal and **(G)** half site view of the patient two months after tumor resection and two stage reconstruction (primary orbital floor and early secondary soft tissue of the upper lip and jaw).

Patient specific implants were covered by autologous soft tissue transplants (see **Figure 8**). Transplants were selected according to the resulting soft tissue deficiency to ensure a sufficient soft tissue coverage of the implant to prevent implant exposure.

### Determination and Definition of the Irradiation Field

The use case of integration of the data of tumor mapping into the irradiation protocol has not yet been established as a clinical routine during the time of the treatment of the 31 CAS patients. However, in 3 cases the information of tumor mapping collected intraoperatively was post-processed and forwarded to the radiation therapist. The data was stored in the preoperative data set in XBrain format. After receipt of the histological findings the virtual points were marked in color depending on the resection status result. The virtual points that were not within the calculated safety distance were also marked in color accordingly. Thus, the radiation therapist could recognize in the data set which points indicated the supposed residual tumor, which points marked an excessively small safety distance and which points were free of tumor tissue.

After the operative reconstruction of the bony midface, the reconstruction result was checked with the help of a postoperative CBCT by default. The XBrain data was merged with the postoperative CBCT data set. Thus, the preoperative data set, the intraoperative tumor marking, the postoperative data set and the tumor mapping, which had been post-processed depending on the histological findings were available to the radiation therapist as an entire DICOM data set for his irradiation field planning. Moreover, the radiation therapist had the option of merging the data made available by the surgeon with a planning CT for irradiation.

### Quality of Life

The quality of life of patients was measured using the University of Washington Quality of Life Questionnaire (UW-QOL v4) (11), a short multifactorial questionnaire that contains specific questions about head and neck tumors. It consists of twelve domains (appearance, activity, recovery, mood, fear, pain, swallowing, chewing, speech, shoulder, taste, saliva). Each of these domains relates to the last seven days and should be rated on a scale that ranges from symptom-free to severe impairment. In addition, the patients should indicate which of these areas was most important to them in the past week. Finally, three global questions were asked about the current state of mind, health-related quality of life and overall quality of life, which were to be answered on a five-point Likert scale. The questionnaire has a high level of validity and is particularly suitable for the questions at hand, as it addresses the specific problems of the patient collective (12, 13).

### Statistical Evaluation

The statistical analyzes were calculated with SPSS for MAC OS X version 22.0 (SPSS Inc., Chicago, IL, USA). The socio-demographic data were analyzed using descriptive statistics. The mean values and standard deviations are given for interval-scaled data and the frequencies are given for ordinal-scaled data. In the case of interval-scaled data, t-tests were calculated for independent samples to compare the mean values. The chi-square test was calculated for the ordinal scaled data. A p-value less than 0.05 was considered significant for all tests.

## Results

### Time for Set-Up of Intraoperative Navigation

**Table 1** gives an overview of the intraoperative navigation times. The registration process in the form of comparing the instruments with the markers took an average of 3.7 minutes with a standard deviation of 1.1 minutes. The duration of the registration depended on the landmarks used. In 22 cases dental splints were used for referencing. Preoperatively intraosseous inserted, screws were used in 9 cases. There was no significant difference in the duration of the registration process comparing dental splints with intraosseous screws, as the screw heads were not covered by skin after preoperative insertion, which abolished the need for exposure. The registration accuracy was measured in millimeters and in the case of the present patient collective was 1.2 mm with a standard deviation of 0.4 mm. The registrations carried out from time to time during the operation to compare the registration accuracy did not show any significant deviations from the original registration accuracy. There was no significant difference in the registration accuracy comparing dental splints with intraosseously inserted screws.

**Table 1:** Time and accuracy of intraoperative navigation.

	M (min)	SD (min)
**time of**		
-**referencing**	3,2	0,3
-**registration**	3,7	1,1
-**intraoperative navigating**	8,8	1,3
-**export of data**	3,9	0,9
**total time of intraoperative navigation**	**19,6**	**2,8**
	**M (mm)**	**SD (mm)**
**accuracy of registration**	**1,2**	**0,4**

Bold values are the main results.

The intra-operative navigation took 8.8 minutes (SD = 1.3 min.), The data transfer to the navigation system including the co-registration with the planning data was carried out automatically and took 3.9 minutes (SD = 0.9 min.).

While set-up of navigation requires a certain amount of time, navigation itself helps to save time during surgery due to the following aspects: improved orientation in the imaging data set, improved selection of the biopsy position and faster surgery due to the real-time navigation feedback. However, the overall saving of time cannot be quantified due to the lack of a comparison of the highly individual cases. Besides assumably shortened operating time CAS offers also advantages concerning quality control due to instant merging of pre- and intraoperative data sets, high consistency between pre-, intra- and postoperative data sets and automatic data saving for postoperative quality control.

### Intraoperative Tumor Marking and Resection

**Table 2** shows the distribution of the descriptive characteristics of the patients with a tumor of the upper jaw treated with CAS as the intervention group compared to those patients treated conventionally without CAS as the control group. The two groups mostly match in their composition. There is no statistically significant difference between the means of the two groups: neither in age calculated by t-test, nor in gender, TNM classification, adjuvant therapy (radiotherapy and chemotherapy), mode of reconstruction or diagnosis checked for by chi-square tests.

**Table 2:** Test characteristics of patients with tumors of the upper jaw treated with the help of CAS in the intervention group compared to the control group treated conventionally without CAS.

	CAS	no CAS (conservatively)	*p*
**age (M ± SD)**	63 ± 17,7	65 ± 10,7	*.350*
**gender (m/f)**	22/9	15/14	*.126*
**pT**			*.688*
** 2**	0	0	
** 3**	9	7	
** 4**	22	22	
**pN**			*.397*
** 0**	23	23	
** 1**	4	5	
** 2**	4	1	
**pM**			
** 0**	0	0	
**R**			*.431*
** 0**	22	16	
** 1**	8	12	
** 2**	1	1	
**radiotherapy (yes/no)**	26/5	19/10	*.100*
**chemotherapy (yes/no)**	8/23	2/27	*.080*
	**CAS**	**no CAS (conservatively)**	***p***
**mode of reconstruction**			*.462*
** obturator prosthesis = no surgical reconstruction**	1	2	
** Latissimus dorsi flap**	16	15	
** Serratus anterior muscle flap**	2	1	
** local primary closure**	4	2	
** upper arm flap**	2	2	
** radial forearm flap**	3	2	
** local flap**	0	4	
** gingiva flap**	0	1	
** Anterolateral thigh flap**	1	0	
** M. temporalis flap**	1	0	
** pedicled palatinal flap**	1	0	
	**CAS**	**no CAS (conservatively)**	***p***
**diagnosis**			*.491*
** squamous cell carcinoma**	19	25	
** adenocystic carcinoma**	4	2	
** mucoepidermoid carcinoma**	1	1	
** osteosarcoma**	1	1	
** ossifying fibroma**	2	0	
** keratocyst**	1	0	
** sinunasal carcinoma**	1	0	
** B-cell non-hodgkin lymphoma**	1	0	
** Ewing’s sarcoma**	1	0	

In the CAS group, 19 (61.3%) patients suffered from squamous cell carcinoma, 4 patients (12.9%) had an adenocystic carcinoma and two patients (6.6%) had an ossifying fibroma. One person each (3.2%) suffered from a keratocyst, a sinunasal carcinoma, a mucoepidemoid carcinoma, a B-cell non-hodgkin lymphoma, an Ewing’s sarcoma or an osteosarcoma as displayed in **Table 2**.

With a number of 16 (51.6%) most patients of the intervention group treated with CAS were reconstructed with a latissimus dorsi flap, three patients (9.7%) were reconstructed with a radial forearm flap and for another four patients (12.9%) a local primary wound closure was sufficient. A serratus anterior muscle flap and an upper arm flap were used in 2 patients (6.5%), respectively. One patient (3.2%) was reconstructed with a temporal muscle flap, one patient (3.2%) with a pedicled palatinal flap and one patient (3,2%) received an anterolateral thigh flap. One of the patients (3.2%) was not reconstructed but received an obturator prosthesis. 8 patients (25.8%) received a chemotherapy after the surgical procedure, 23 patients (74.2%) a radiotherapy.

**Figure 9:**
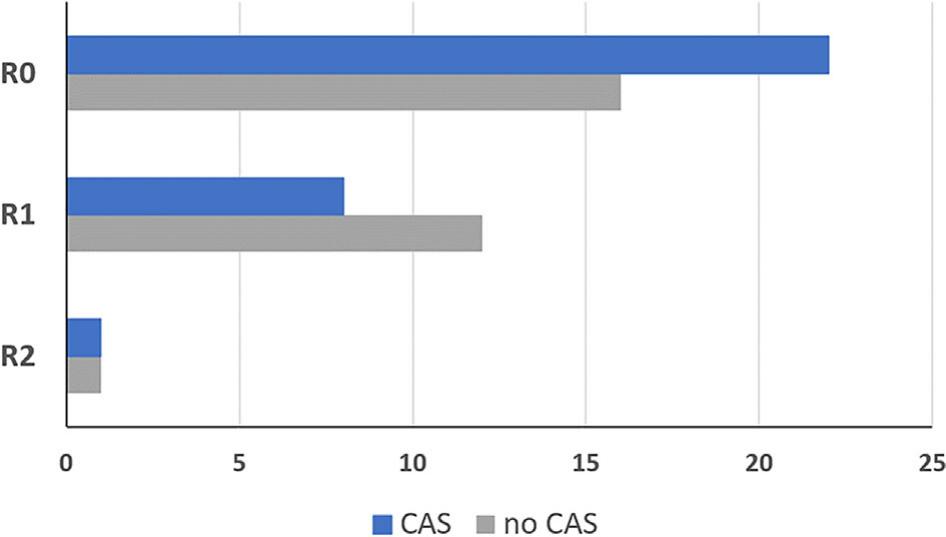
Resection result of the group that was treated using the CAS, compared to the control group. R0: No tumor detectable in the organism R1: Microscopic residual tumor at the resection border R2: Macroscopically tumor or metastases remaining in the patient. Dark blue = intervention group treated with CAS, grey = control group treated without CAS.

In the CAS group, frozen sections samples were collected in higher numbers and smaller sizes compared to the control group. An average of 15.3 frozen sections were collected per CAS patient with an average size of 8.7 mm. In the conventional group an average of 5.3 frozen sections were collected per patient with an average size of 23.2 mm. More R0 resections were achieved in patients treated with CAS in the intervention group compared with the control group as displayed in **Figure 9**. In the CAS group, an R0 resection could be performed in 22 patients (71.0%), while in the control group there were 16 patients (55.2%). An R1 resection was performed in 8 patients (25.8%) in the CAS group compared to 12 patients (41.4%) in the control group. In both groups one patient showed an R2 resection (CAS = 3.2%; control group = 3.4%), which was performed because the tumor had infiltrated the internal carotid artery as a vital structure. However, the differences are not significant (df = 58; t = -1.112; p = 0.271).

### Reconstruction After Ablative Surgery

Resection defects after the loss of hard and soft tissue due to ablative surgery of the midface result in considerable functional and aesthetic deficits. These changes are even more dramatic if the orbit is affected by the resection. Once the orbital bony boundaries and/or the intra-orbital soft tissues are impaired, the resulting imbalance between the eyeball and the supporting structures lead to symptoms such as enophthalmus, double vision and facial asymmetry. This particularly applies for orbital prosthesis in cases of anophthalmic patients. Reconstruction of these cases involves a repositioning of the eye globe/prosthesis which might need a reduction of the intra-orbital volume due to intra-orbital soft tissue loss achieved by a reforming of the boundaries of the orbital cavity. Due to the intricate three-dimensional anatomy and the wish for a better prediction of the result, it is advisable to use modern principles of CAS in these cases. CAS allows a backwards planning, which enables the virtual planning of the desired position of the eyeball/eye prosthesis first followed by the calculation of the necessary intra-orbital volume reduction and thus the needed recontouring of the orbital boundaries. This recontouring is achieved *via* rapid prototyping and prebenting of titanium meshes. The anatomically correct positioning is monitored *via* intraoperative navigation. The extra-orbital soft tissue defect can be replaced *via* autologous tissue transfer.

**Figure 10:**
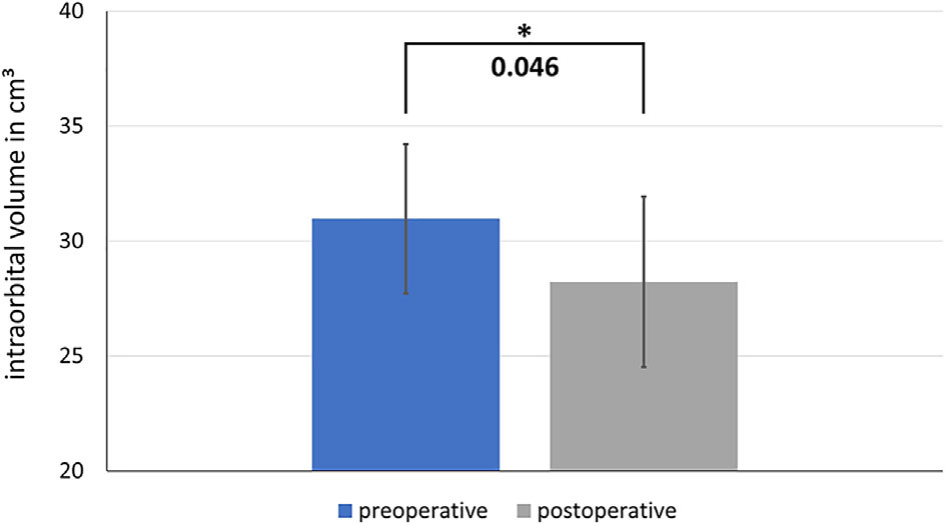
Comparison of the pre- and postoperative intraorbital volumes after ablative surgery and primary reconstruction using CAS. The difference between the preoperative and postoperative volume is significant. * shows significance with a p-value < 0.05

Here we present data of 15 primary cases in need of midfacial reconstruction after ablative tumor surgery involving the orbit. Pre-operative planning took 25 min for the surgeon and 60 min for the technical staff on average, while manufacturing of the model and the implant took 2 working days. **Table 3** compares the pre- and postoperative volumes of these patients after ablative surgery using CAS. In all cases the intra-orbital volume was reduced, which led to a more symmetrical positioning of and improved support for the eye globe/prosthesis and thereby for the accompanying structures like the eye lids.The mean preoperative volume for primary reconstructions was 30.97 cm^3^ (SD = 3.25 cm^3^) and the postoperative volume was 28.23 cm^3^ (SD = 3.70 cm^3^). A t-test for independent samples showed that the mean difference between the pre- and postoperative volumes was significant (df = 28; t = 2.085; p = 0.046) (see **Figure 10**).

**Table 3:** Pre- and postoperative volumes of the patients with a primary reconstruction of the orbit using the CAS.

patient number	affected site	preoperative volume (cm^3^)	postoperative volume (cm^3^)
**primary reconstruction**			
** 1**	r	30.900	23.642
** 2**	r	27.777	24.128
** 3**	l	29.826	27.174
** 4**	l	32.193	30.987
** 5**	r	30.038	24.878
** 6**	l	28.396	25.656
** 7**	r	31.971	28.624
** 8**	l	30.378	28.375
** 9**	l	39.378	36.895
** 10**	r	31.407	29.343
** 11**	r	34.535	32.535
** 12**	r	25.073	23.488
** 13**	l	33.258	32.360
** 14**	l	27.517	25.912
** 15**	l	31.960	29.423
** mean**	***p = 0.046***	**30.97**	**28.23**
** standard deviation**		**3.25**	**3.70**

Bold values are the main results.

### Optimization of the Interface Between Surgery and Radiation Therapy Using CAPP and CAS

So far, only the treatment results of three patients could be evaluated, because although the data could be passed on from the surgeon to the radiation therapist, the radiation therapists do not yet routinely include this information, but rather use their planning CTs as a basis for their therapy. However, since the first results are very promising, our efforts are aimed at routinely providing the protocols, which were mainly prepared with the help of the CAS, to the radiotherapists in order to use these protocols as a basis for their therapy. We assume that this procedure will gain a foothold in routine clinical practice. Since this method is still new, the post-processing of the data for passing on to the radiation therapist currently still takes some time. According to previous experiences, this takes about 10 minutes.

### Case Study of Selective Irradiation After Virtual Tumor Marking With the Help of CAS

With the help of this case study, the advantages of intraoperative data collection and transfer to the radiation therapists are explained. A 67-year-old patient with a primary SCC of the left maxilla invading the orbital floor (cT4) was operated on using CAS. Intraoperative, navigation-assisted tumor mapping and tumor resection were used. The histopathological findings near the base of the skull still showed parts of a residual tumor in two localizations of the tumor map, there were no close margin areas. This information was highlighted in color in the intra-operative tumor map.

Due to the location of the residual and the reduced general health condition of the patient a further resection could not be performed. Therefore, the files were passed on to the radiation therapist in DICOM format. The iPlan 4.0 beta software was used. Only those coordinates (voxels) that represented a positive histology or a close-margin resection were marked with the Houndsfield unit of 3500 H. This value has been chosen well above the usual maximum limit of 3100 H to ensure that the original DICOM data record could not be manipulated any further. This provided another advantage as the marked points could be quickly found again using the threshold algorithm. This improved data set was exported as “enhanced DICOM format” (enDICOM) and could be imported into common irradiation devices.

In this case, the enDICOM data set was imported in the Oncentra MasterPlan System (version 3.3, Nucletron, Netherlands/USA) and co-registered with the postoperative radiation therapy planning CT scan. With this additional information, a more selective irradiation of the midface resulting in smaller volumes of high dose irradiation could be guaranteed.

### Quality of Life

For the calculation, the responses of the intervention group that were treated using CAS for tumors of the upper jaw were compared to the responses from the control group which were treated conventionally without CAS. A mean value comparison was calculated using the t-test for independent samples. Out of the included 60 patients a total of 42 patients fully completed and returned the QoL-questionnaire and thereby were included in the calculation. The group that was treated using CAS had a response rate of 68% and the control group a response rate of 72% which results in 21 patients of each group.

**Figure 11:**
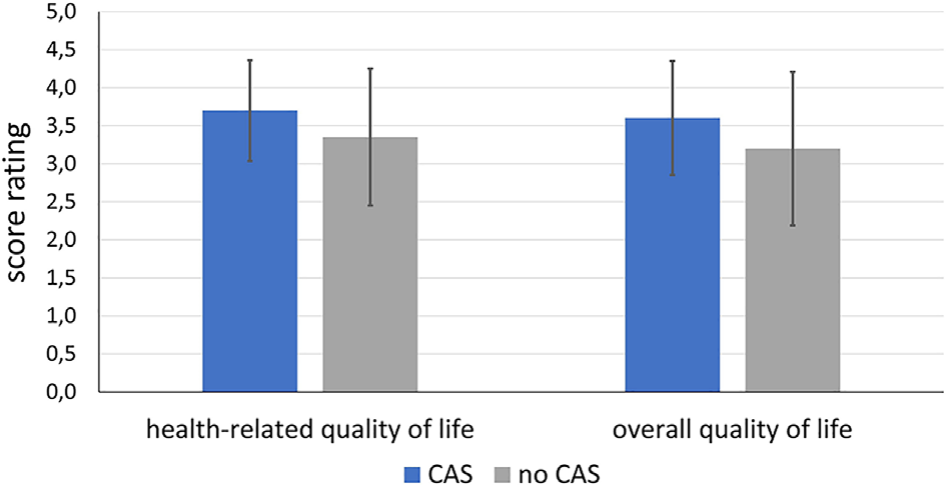
Comparison of health-related and overall quality of life of the patients of the intervention group treated using CAS compared to the control group treated conventionally without CAS.

**Figure 11** shows the distribution of the score rating with regard to the global question about health-related quality of life and overall quality of life. For both, the CAS group tends to have a better scoring (health-related quality of life: M = 3.70; SD = 0.66, overall quality of life: M = 3.60; SD = 0.75) than the control group (health-related quality of life: M = 3.35; SD = 0.90, overall quality of life: M = 3.20; SD = 1.01). However, the differences do not become significant neither in terms of health-related quality of life (df = 38; t = 1.872; p = 0.069) nor for the overall quality of life (df = 38; t = 1.424; p = 0.163).

When looking at the question of how the patients are doing compared to the months prior to diagnosis, the group treated with CAS (M = 2.80; SD = 1.19) tended to have better scores than the control group (M = 2.65; SD = 1.23) without the difference becoming significant (df = 38; t = -0.392; p = 0.698).

**Figure 12:**
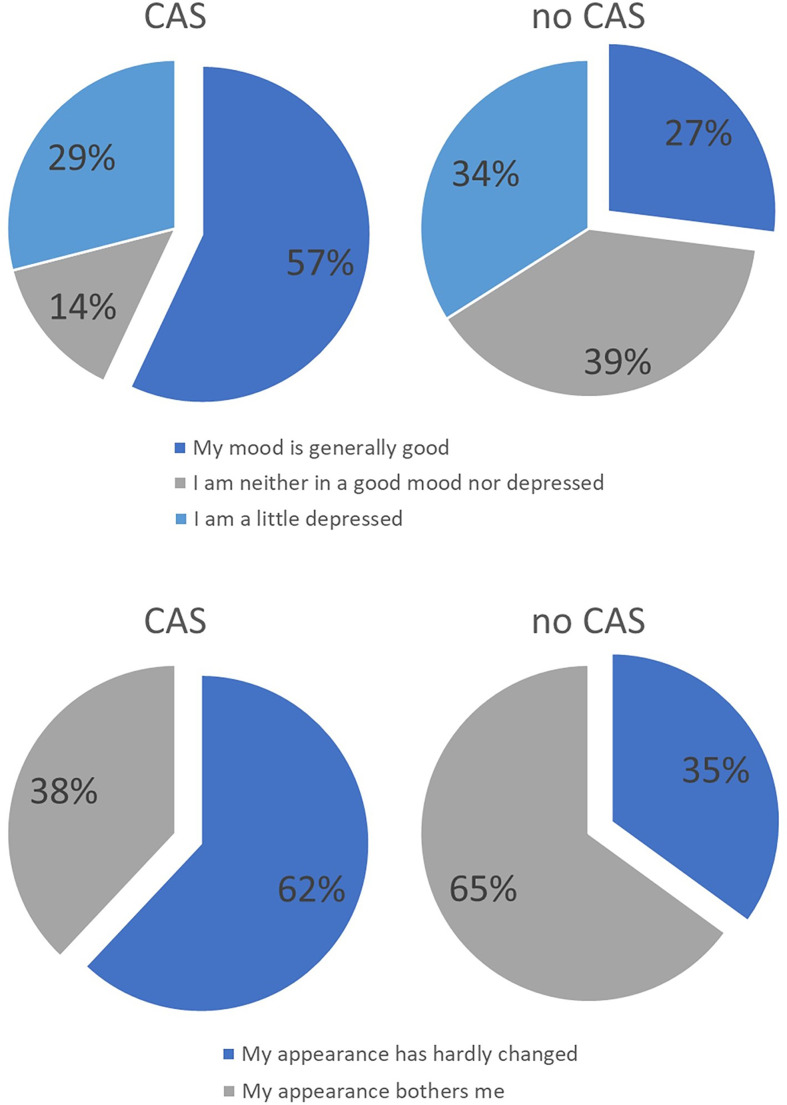
Distribution of answers of the intervention group treated with CAS compared to the control group treated without CAS. In the upper part distribution of answers regarding the question of the mood in recent weeks are displayed while in the lower part distribution of answers regarding the question of ‘how you feel about your own external appearance in recent weeks’ are shown.

With regard to the domains, it was found that there tended to be differences in the areas of mood and appearance. In the control group, more patients stated that they were bothered by their appearance and that their mood was impaired. There were no differences between the groups with regard to the other domains. **Figure 12** compares the results of both groups with regard to the variables appearance and mood. For the sake of clarity, the items have been combined into two or three statements.

## Discussion

The routine use of CAPP and CAS in the treatment of upper and lower jaw tumors in the maxillofacial surgery was often prevented by the high costs and poor user friendliness due to the high technical complexity and the demanding operation of the systems. Regarding the costs we have the beneficial situation in Germany that the total use of computer-assisted surgery and orbital reconstruction is being reimbursed because of many publications and the clear consensus in the indication having the benefit of precise reconstruction, excellent surgical results and saving time in terms of operation hours.However, there is a need for a comprehensive system with which the entire procedure, from planning to implementation and result control, can be carried out efficiently and easily in order to optimize patient treatment and reduce overall costs. In the present work such a method for the therapy optimization of tumors of the upper jaw and midface area with the necessary treatment steps was presented and evaluated based on the analysis of 31 patients with a tumor of the midface and their surgical treatment with the help of CAS. The preoperative planning, intraoperative navigation with navigated tumor resection and tumor mapping, the reconstruction after ablative surgery and optimization of the interface between surgery and radiation therapy were examined in detail. The CAPP and CAS introduce a new level of quality control and quality assurance and thus promote the professionalization of surgery, because the success of the treatment can now be quantified. From a medico-legal perspective, the surgeon can protect himself externally with the CAPP and CAS, since the documentation of the individual operative sub-steps makes all therapeutic measures transparent, traceable and verifiable at all times. At the same time, the demands on one’s own performance increase because the intervention is no longer carried out intuitively or on the basis of experience. Due to the technical possibilities, the aim is to plan and implement the intervention down to the millimeter.

Advantages of these procedures has been presented before (14–18). The current study provides data of a prospective clinical study including 31 patients with midfacial tumors treated with CAS in comparison with 29 patients treated conventionally.

### Intraoperative Navigation

The steps associated with intraoperative navigation such as referencing, registration, intraoperative navigation and data transfer took an average of 19.6 minutes (SD = 2.8 minutes). In order to be able to decide whether this additional effort pays off in terms of a cost-benefit ratio, the additional time effort must be compared with indicators of the benefit. Initial studies show that the additional time invested of around 30 minutes predominates (19, 20) and that the more experienced the user is with the method of navigation, the quicker the intra-operative set-up of navigation devices (21). First of all, the operating time itself is important, but it is difficult to quantify objectively, as the operating time depends on the patient’s individual clinical situation. However, there are already studies that have shown a shortening of the operating time (22, 23). In addition to the time saved, the qualitative benefit for the patient should also be taken into account (24). With regard to the time savings, it should be remembered that the detailed preparation for the operation means that all structures are known and that better orientation in the image data set is possible (25). Due to the prior planning and virtual simulation of the access routes, the best access route including alternatives is determined before the operation, so that targeted control of the desired structures is possible, which not only makes the intervention safer, but also reaches the goal faster (26). The reconstruction does not have to be carried out according to the time-consuming “trial and error” procedure in which the best position for the implant has to be selected (27, 28). By planning and adapting the implant beforehand using rapid prototyping, the correct implant position can be controlled directly and immediately adapted using the feedback from the intraoperative navigation (29). It is also positive that the patient is not exposed to further radiation during surgery due to the x-rayless navigation system. A high degree of consistency between the pre-, intra- and postoperative data evaluated through the merging of the data sets allows a statement to be made about the success of the intervention and thus makes the procedure more transparent for all involved (30–32). By storing all the data sets, the patient has the benefit of a complete treatment plan, since the radiation therapist can also benefit from the data and can plan his therapy precisely tailored to the patient (33).

### Navigation-Assisted Tumor Resection and Tumor Mapping

A considerable problem of conventional tumor resection without navigation is that the anatomical three-dimensional position of the specimen is described in words written down in the surgery report and in the pathology request document by the operating surgeon. The achieved accuracy can hardly be compared to the method of navigated tumor resection and tumor mapping. This method links the specimen to a coordinate in the three-dimensional anatomy of the patient. Technically this coordinate represents one precise point in space which refers to a sphere-shaped volume with a radius of 1.2 mm due to the measured registration accuracy during navigation. With a mean specimen size of the taken resection margins of 8.7 mm the virtual coordinate does not congruently match in full size. However, by taking at least 12 specimen the outline of the resection margin is adequately captured. Together with the histopathological result, this serves as an objective virtual marking of the true tumor extent. This higher number of taken specimen seems only applicable with the help of CAS since differentiation during relocation cannot be achieved with conventional location- and orientation-labeling by simple word description. The orientation-labeling and achieved accuracy of relocation with intraoperative navigation method adequately serves the technical achievable accuracy especially in cases of re-resection and should lie above the accuracy of the previous used conventional method. Even in view of soft tissue shifting presenting a well-known problem with intraoperative navigation (24, 34, 35), the presented method of mapping of midfacial tumors seems still applicable because in the anatomical region of the midface there are many bony structures giving stability to the adjacent soft tissue and thereby limiting the bias of soft tissue shifting.

In combination with the preoperative virtual planning of the resection extent which relies on merging of different imaging modalities combining the different information of these modalities (36), this method of navigated tumor resection and tumor mapping harbors promising results. In our study, we showed that with this method there were more R0 resection possible within tumors of the upper jaw compared to the conventional tumor resection. However, the difference between the two groups was not significant probably due to the sample size. It seems debatable that the observed increase in R0 resections in the CAS group may be achieved by the higher number of frozen sections rather than the use of navigation and CAS. However, in our opinion this high number of frozen sections cannot be adequately labeled with conventional methods allowing precise relocation in case of re-resection. Therefore, a technically applicable sampling of a high number of frozen sections is only reasonably achievable through CAS as already stated above. The comparison of the groups with regard to age, gender, radiation, diagnosis and reconstruction method shows that the groups did not differ in any respect. The CAS is therefore not only indicated for certain patients but can be used in all cases in everyday clinical practice. It has been proven to be particularly advantageous in the case of T4-tumors, since here, a successful operation without the use of CAS in terms of free resection margins is less likely (37, 38).

Even though no clear statements can be made about the long-term success of the CAS compared to previous methods, it should be noted that the CAS makes it easier to maintain the safety margin (37–39). Longitudinal studies will show whether the recurrence rate can be reduced in the long term, as this is already the case in other disciplines (40–42).

Another advantage of the CAS is that all information about the patient’s anatomy is available preoperatively. This means that it is known how far the safety distance can be maintained before the intervention. If, for example, the safety margin touches the base of the skull, bony structures can be removed or preserved with greater certainty, since the distance to intracranial perforation is known. Our experience has shown that CAS has proven itself particularly well in the midface area, since most tumors are closely related to bony structures. This is precisely why the CAS is particularly suitable for T3 and T4 tumors.

### Reconstruction After Ablative Surgery

The loss of hard and soft tissue after tumor resection is associated with substantial functional and aesthetic deficits (43). CAS in the form of computer-assisted design and models (CAD/CAM) has by far improved the possibilities of reconstructive surgery, especially in the planning of symmetrical aspects of the bony contour (28, 44–47). With regard to the primary reconstruction of the midface, the focus of the functional and aesthetic rehabilitation is, in addition to the chewing function, on the reconstruction of the orbital region. Since the secondary reconstruction can be expected to show poorer functional and aesthetic results due to various challenges, the focus should be on the primary reconstruction, in which the implants are inserted directly after the tumor resection. In doing so, not only the resection but also the implant insertion is carried out with the help of a template, since the position of the implant can be planned preoperatively according to the prosthetic situation. With the rapid prototyping or computer-assisted design/modeling (CAD/CAM) process, it is possible to create a virtual template for unilateral defects using simple processes such as mirroring the unaffected side onto the affected side, through which the midface can be restored (3). If there are bilateral defects, there is also the option of importing the midface from the atlas segmentation and adapting it to the remaining bony structures (elastic and rigid deformation) (48). This is used, for example, for complex tumors of the midface. Once the tumor has been resected, the periorbital region is reconstructed with alloplastic materials and autologous bone grafts. Various CAS procedures can be used here.

These procedures have opened up new possibilities and also a new claim for reconstructive surgery of the facial skull since it is now possible to achieve better aesthetic results through the symmetrical and thus faithful restoration of the removed structures. While prior to the introduction of these methods it was primarily a matter of repositioning the vascular or avascular bone in such a way that functionality was restored, the steps necessary to completely restore the face can now be planned preoperatively, taking into account the aesthetic subtleties (44, 45).

Our data showed that when the orbit is reconstructed after tumor surgery using CAS, the postoperative volume decreases. The postoperative reduction in volume is needed in cases of intra-orbital soft tissue deficiencies. Moreover, potential soft tissue atrophy and scaring may occur after surgery in this area which can cause enophthalmus. Therefore, we preferred a slight undercorrection rather than an overcorrection But even if the intra-orbital soft tissue was not touched, the postoperative volume was decreased. On the one hand, this is due to the fact, that a new implant is inserted which has its own volume, which must be taken into account. The segmented orbit mesh has an average volume of about 1.3 cm^3^. If this number was added to the postoperative orbital volume, the difference between the pre- and postoperative volumes would no longer be significant. Deviations are also more likely with regard to the orbit compared to midface reconstruction since the structures are more filigree and complex and manual adjustments are therefore often necessary. On the other hand, the orbit mesh is bent manually using the STL models, so that minor errors can occur.

### Successful Irradiation With Computer-Assisted Surgery

According to Boehm et al. only a few technical solutions for structured data storage and processing of all patient-related data are scientifically described (49). As already described above, after an operation it is necessary to transmit the data to the radiation therapist so that he can optimally plan the therapy. Without the CAS, the radiation therapist has insufficient information available in the form of histological findings, surgical reports and CT data sets, according to which he must plan a strategy. With the help of the CAS, screenshots in form of jpeg files and even enhanced DICOM data sets can be transmitted to the pathologist after the surgical procedure, so that the information can be passed on in a language-independent and unambiguous manner. The 3D volume rendering enables the pathologist to identify the location of the tumor in the image. Based on the histological results, positive boundaries can then be marked in the data set or drawn in in color. As part of further oncological treatment, this data can be saved and transmitted in DICOM format to oncologists, radiologists and pathologists so that they can receive specific tumor information such as the invasion of adjacent structures. Therefore, CAS can serve to enhance the interdisciplinary collaboration and improve patients’ outcome. This is especially relevant for the treatment planning of adjuvant radiation therapy. While radiation therapy has been shown to reduce loco-regional recurrence rates (50), it harbors significant side effects such as xerostomia. These side effects occur due to the damaging of healthy tissue surrounding the tumor tissue. The planning of dose distribution during radiation relies on post-operative imaging (51). However, it can be challenging to differentiate on the basis of the imaging data between tissue aberrations due to remaining tumor or due to postoperative changes. The described procedure of tumor mapping can link the pathological information to a definitive three-dimensional coordinate and thereby helps reduce any doubt during interpretation of the imaging data. This allows a more precise dose distribution. However, if this procedure leads to a further reduced loco-regional recurrence rate or to lesser side effects during radiation therapy remains to be seen in further studies focusing on statistically representative patient groups which underwent adjuvant radiation therapy relying on CAS.

### Quality of Life

It is difficult to quantify patients’ quality of life in terms of CAS due to various problems. On the one hand, due to the lack of a comparison option, the patient is often not even aware that the CAS, for example, was able to remove the tumor more effectively or that vital structures could be better preserved. In addition due to the fact that patients are suffering from cancer and have to undergo an operation, the level of suffering among patients is so high that the quality of life is impaired for all patients, whether they were operated with or without the use of CAS (52–54). Large numbers of cases would be necessary to calculate significant differences between the groups. However, our data showed that those patients who were operated on using CAS had higher scores, especially in the areas of mood and appearance. These factors in turn correlate with one another.

When asked about the worst event since the operation, patients with oral cancer above all mentioned the realization of facial distortions, even if these seemed minor to third parties (55, 56). This shows that the patient’s appearance is particularly important, which is understandable in terms of social integration after treatment. Appearance is also related to mood. While in the past the patient’s survival was in the foreground and the operation was therefore extremely radical, CAS offers far more options here. By setting a safety distance in advance as part of the preoperative planning, it becomes evident which structures need to be removed, so that the reconstruction of the face can be planned in detail in advance and patient-specific implants can be made. Therefore, there was a tendency towards a higher health-related and overall quality of life, on which the factors appearance and mood exert a decisive influence. In particular for health-related quality of life, our results became almost significant, which suggests that a larger sample might show significant results.

## Conclusion

The CAS offers great advantages over the previously established therapy methods in ablative tumor surgery, which are primarily reflected in the precision, safety and success of the treatment. Thanks to sophisticated systems for merging data sets from different imaging processes and the resulting possibilities for precise preoperative planning, in which the treatment goal can be determined in advance, taking into account all the necessary information, the operation can be less invasive while still maintaining a successful and more predictable result. Moreover, the possibility of computer-assisted implementation and documentation enables interdisciplinary, language-independent and thus unambiguous communication due to the broad database. Given these advantages of CAS, it is surprising that CAS is not yet routinely used in clinics. Due to the costs and the time required for the implementation and familiarization with the systems it becomes understandable that some surgeons have not yet been convinced of the use of the CAS.

In this study, we showed distinct advantages of CAS in the complex oncologic surgery of the midface. The operation and the planned expected results of the operation can be simulated preoperatively on the computer, afterwards an intraoperative implementation of the simulation is possible with the help of the navigation. The postoperative control through image fusion of the preoperative plan with a postoperative data set enables an evaluation of the reconstruction result with millimeter precision, as it is desired for complex reconstructions.

Another great advantage of this system is that all steps in the diagnosis and therapy of the patient are traceable at any time. If surgical obstacles arise intraoperatively, an alternative procedure with this system can be carried out with a minimum of effort and the complete tumor resection can still be ensured. Thus, the goal of improving the success of the treatment, optimization of interdisciplinary collaboration and the patient’s quality of life through complete tumor resection and adequate reconstruction is retained.

## Data Availability Statement

The raw data supporting the conclusions of this article will be made available by the authors, without undue reservation.

## Ethics Statement

The studies involving human participants were reviewed and approved by Ethics committee of Medical School of Hannover. The patients/participants provided their written informed consent to participate in this study. Written informed consent was obtained from the individual(s) for the publication of any potentially identifiable images or data included in this article.

## Author Contributions

MR conceived of the work and participated in its design and coordination. MW and MR made substantial contributions to data acquisition and conception of manuscript. MW drafted and designed the manuscript. MR has been involved in drafting and revising the manuscript. All authors contributed to the article and approved the submitted version.

## Conflict of Interest

The authors declare that the research was conducted in the absence of any commercial or financial relationships that could be construed as a potential conflict of interest.

## Publisher’s Note

All claims expressed in this article are solely those of the authors and do not necessarily represent those of their affiliated organizations, or those of the publisher, the editors and the reviewers. Any product that may be evaluated in this article, or claim that may be made by its manufacturer, is not guaranteed or endorsed by the publisher.
